# Supplementary material for: Using epidemic simulators for monitoring an ongoing epidemic
Source: Sci Rep. 2020 Oct 6;10:16571. doi: 10.1038/s41598-020-73308-5 (PMC7538994; doi:10.1038/s41598-020-73308-5)
Supplement: Supplementary file 1 — Supplementary Information. [file 41598_2020_73308_MOESM1_ESM.pdf]

Using epidemic simulators for monitoring an ongoing epidemic

Raghavan, M[1] **Sridharan, K.S.**[1] **Mandayam, Y.R.**[1]
[1]*Indian Institute of Technology - Hyderabad, Dept. of Biomedical engineering, Hyderabad, 502285, India*

Supplementary material

Modelling equations

E, I, R: Fraction of population that are exposed, undetected infections or recoveries, *no unit*.
Eq, Iq, Rq: Corresponding fraction of populations that are quarantined, *no unit*.
D: Fatalities from the undetected population(as a fraction of total population), but they may be detected close to death or post-mortem, *no unit*.
D_q: Fatalities from quarantined infections(as a fraction of total population), *no unit*.
a: Fraction of Infected that acquired it by direct contact with another infected, *no unit*.
c: Fraction of exposed or contact infected individuals that are detected, *no unit*.
q: Fraction of community infected that are detected by self reporting or random testing, *no unit*.
δ: Fraction of infected that succumb to the disease, *no unit*.
γ: Inverse of the mean time of infection, day^{-1}.
k: Inverse of mean latency time, day^{-1}.
β: Transmission rate given by product of contact rate and probability of infecting a contact, day^{-1}.
ϕ: Rate of influx in persons / day (normalised by total population), day^{-1}.
p_I: Probability that an incomer is infected or exposed, *no unit*.

$$\frac{dS}{dt} = -\beta SI + \phi(1 - p_I)$$

$$\frac{dE}{dt} = \beta SI\ (1 - c) - kE + \frac{\phi p_I}{2}$$

$$\frac{dI}{dt} = kE\ \{a(1 - c) + (1 - a)(1 - q)\} - \gamma I + \frac{\phi p_I}{2}$$

$$\frac{dR}{dt} = \gamma\,(1 - \delta) I$$

$$\frac{dD}{dt} = \gamma\,\delta I$$

$$\frac{dE_q}{dt} = \beta SI\ c - kE_q$$

$$\frac{dI_q}{dt} = kE\{ac + (1-a)q\} + kE_q - \gamma\, I_q$$

$$\frac{dR_q}{dt} = \gamma\,(1-\delta)\; I_q$$

$$\frac{dD_q}{dt} = \gamma\;\delta\; I_q$$

Model parameters for states

State	Fatality rate	Paramters at t=0								Parameters at other time points		
		Beta	c	q	Lag of recovery	Population	Influx population (in_phi)	p (influx infected)	Pre-start	Time point	Parameter	New value
MH	7%	5.7/9	0.50	0.10	8	114200000	100	0.10	7	8-Mar-2020	c	0.1
										20-Mar-2020	in_phi	0
										30-Mar-2020	c	0.25
										14-Apr-2020	c	0.1
GJ	7%	4/9	0.50	0.20	8	62700000	0	0.10	0	18-Mar-2020	in_phi	180
										22-Mar-2020	in_phi	0
										4-Apr-2020	beta	8.4/9
										4-Apr-2020	in_phi	410
										4-Apr-2020	c	0.25
										14-Apr-2020	in_phi	0
DL	4%	4/9	0.10	0.30	8	21800000	5	0.10	15	27-Feb-2020	in_phi	40
										15-Mar-2020	c	0.45
										18-Mar-2020	in_phi	0
										27-Mar-2020	in_phi	450
										29-Mar-2020	c	0.75
										9-Apr-2020	in_phi	0
KA	5%	5/9	0.10	0.10	8	64100000	5	0.01	20	24-Feb-2020	c	0.4
										15-Mar-2020	in_phi	150
										29-Mar-2020	in_phi	0
										15-Apr-2020	c	0.95
RJ	2%	4.5/9	0.20	0.10	8	68900000	0	0.10	4	9-Mar-2020	c	0.5
										13-Mar-2020	in_phi	48
										25-Mar-2020	beta	8/9
										30-Mar-2020	in_phi	0
										10-Apr-2020	c	75
TN	1.50%	4/9	0.50	0.40	7	67900000	0	0.10	-1	20-Mar-2020	in_phi	50
										27-Mar-2020	in_phi	1200
										29-Mar-2020	beta	2/9
										29-Mar-2020	c	0.8
										5-Apr-2020	in_phi	0
MP	8%	8.55/9	0.10	0.10	8	73300000	0	0.10	5	9-Mar-2020	in_phi	25
										19-Mar-2020	in_phi	1
										25-Mar-2020	c	0.35
										14-Apr-2020	c	0.9
KL	1%	3.06/9	0.20	0.30	6	34800000	100	0.10	10	10-Mar-2020	in_phi	1
										25-Mar-2020	beta	43839
										25-Mar-2020	c	0.6
WB	7%	5.3/9	0.20	0.30	8	90300000	0	0.10	0	15-Mar-2020	in_phi	40
										20-Mar-2020	in_phi	0
TG	4%	4.5/9	0.30	0.30	8	35200000	5	0.10	4	14-Mar-2020	in_phi	80
										24-Mar-2020	in_phi	0
										31-Mar-2020	in_phi	450
										31-Mar-2020	c	0.7
										4-Apr-2020	in_phi	0
PB	8%	4/9	0.00	0.10	8	28000000	0	0.10	2	12-Mar-2020	in_phi	40
										19-Mar-2020	c	0.7
										20-Mar-2020	in_phi	0
										4-Apr-2020	in_phi	190
										10-Apr-2020	in_phi	0
UP	3%	4.6/9	0.10	0.10	5	204200000	0	0.10	18	10-Mar-2020	c	0.2

Table 1. **(supplementary) Model parameters for various states:** The values of all the model parameters used along with temporal variations are listed.
